# Glucosamine Inhibits the Proliferation of Hepatocellular Carcinoma Cells by Eliciting Apoptosis, Autophagy, and the Anti-Warburg Effect

**DOI:** 10.1155/sci5/5685884

**Published:** 2025-01-08

**Authors:** Misako Samizu, Kaoruko Iida

**Affiliations:** ^1^Department of Food and Nutritional Sciences, Graduate School of Humanities and Sciences, Ochanomizu University, Tokyo 1128610, Japan; ^2^Division of Nutritional Science, Institute of Human Life Science, Ochanomizu University, Tokyo 1128610, Japan

**Keywords:** aerobic glycolysis, apoptosis, autophagy, glucosamine, hepatocellular carcinoma

## Abstract

Although glucosamine (GlcN) exhibits antitumor effects, its mechanism of action remains controversial. Additionally, its impact on hepatocellular carcinoma (HCC) is not well understood. This study aimed to investigate the antitumor effects of GlcN and its underlying mechanism in a mouse HCC cell line, Hepa1-6. GlcN treatment significantly inhibited Hepa1-6 cell proliferation. Gene expression analysis revealed that GlcN upregulated *Chop* and *Bax* while downregulating *Bcl2*, indicating the involvement of endoplasmic reticulum (ER) stress-induced apoptosis in the antiproliferative effects of GlcN. GlcN also increased the expression of *FoxO1* and *FoxO3*, known tumor suppressors in various cancers. Furthermore, GlcN treatment elevated the levels of LC3II (an autophagy marker) and AMP-activated protein kinase activity, suggesting intracellular energy shortage. Indeed, GlcN treatment significantly suppressed glycolytic flux, lactate, and ATP production. Supplementing GlcN treatment with a high glucose concentration (20 mM) significantly attenuated its effect. We postulate that GlcN inhibits Hepa1-6 cell growth by inducing ER stress-induced apoptosis and autophagy and by inhibiting aerobic glycolysis (the Warburg effect), a key hallmark of cancer metabolism. Given that glucose transporter 2 (GLUT2), which is abundantly expressed in hepatocytes, has a high affinity for GlcN, these effects may result from GlcN competing with glucose for hepatocyte uptake by GLUT2. Our novel findings have potential implications for HCC treatment.

## 1. Introduction

Glucosamine (GlcN) is the most common amino sugar found in nature. It is present in human joint cartilage and skin; in crustacean shells, it occurs as various polysaccharides, such as glycosaminoglycans and chitin. GlcN has been widely used as a dietary supplement for decades, and numerous clinical studies have investigated its pharmacokinetic and physiological effects, primarily focusing on its use in osteoarthritis [1–3]. The proposed mechanism underlying the therapeutic effects of GlcN involves anti-inflammatory activity by inhibiting nuclear factor kappa B signaling [4]. Recent studies have suggested that the antioxidant activity of GlcN may confer additional benefits, such as cardio- and neuroprotection [4], although these findings remain controversial.

In addition to these effects, GlcN reportedly possesses anticancer properties. Approximately 70 years ago, intraperitoneal administration of GlcN was first reported to exhibit specific cytotoxicity against sarcoma transplanted in mice, improving their hepatic catalase activity [5]. Another classical study involving rats bearing Walker's tumors showed that intravenous GlcN administration for up to 40 h [6] resulted in complete tumor cell necrosis, whereas healthy hepatic and renal cells only experienced transient morphological changes. The anticancer effects of GlcN have been observed in various tumor cell lines derived from human and other mammalian tumors, with different mechanisms proposed for this phenomenon [7]. Furthermore, recent large prospective cohort studies have shown an association between GlcN use and a lower incidence of and mortality from specific cancers, such as kidney, lung, and rectal cancer [8–10]. In contrast, another study claimed that regular GlcN use was associated with an equal or even higher risk of almost all the cancer types analyzed [11].

These inconsistent results may partly arise from the difference in GlcN bioavailability in each cancer type. When taken orally, 90% of GlcN is absorbed but rapidly metabolized by the liver, resulting in relatively low bioavailability (only 26% of that following intravenous administration) [12, 13]. Thus, oral administration of GlcN at a regular dosage only achieves a slightly higher serum concentration of approximately 0.06 mM as compared to the ∼0.04 mM present without administration [14]. Although the achievable blood concentration of GlcN through oral administration is limited, it can accumulate in specific organs, including the liver, kidneys, and articular cartilage, during its metabolic process [15]. GlcN also has the advantage of high specificity for malignant cells, with GlcN-conjugated drugs exhibiting higher specificity for malignant cells than their unconjugated forms [16–18]. This property seems to be derived from the interaction of GlcN with glucose transporters (GLUTs) [17, 18]; however, the types and expression levels of GLUTs differ among tumor cell types, generally depending on their organ of origin.

Hepatocellular carcinoma (HCC) accounts for the majority of liver cancer and is a leading cause of mortality among various cancer types. Like many other cancers, HCC preferentially uses glucose; however, it also exhibits a unique feature that could counteract this metabolic action. GLUT2, which has a relatively low affinity for glucose, is abundantly expressed in HCC [19, 20]. This could be attributed to the physiological role of the liver, from which HCC develops. GLUT2 is the major transporter isoform expressed in the adult liver, enabling the organ to absorb and metabolize glucose when blood glucose levels are high, storing excess glucose as glycogen. Additionally, GLUT2 has a much greater affinity for GlcN than for glucose at physiological concentrations (∼5 mM) [21]. Thus, GlcN may be preferentially taken up by HCC tissues and act as a potent agent for HCC treatment. Nevertheless, very limited research has focused on the effects of GlcN on HCC, except for one study that described a toxic effect of GlcN on a human HCC cell line (SMMX-7721) via cell cycle arrest [22].

In this study, we aimed to address this research gap and elucidate the anticancer potential of GlcN on HCC. Specifically, our objective was to assess the molecular mechanisms underlying GlcN action in this context using a mouse HCC cell line (Hepa1-6). Our findings will contribute substantially to the development of novel treatments for HCC using the therapeutic application of this compound.

## 2. Materials and Methods

### 2.1. Cell Culture and Treatment

D-(+)-Glucosamine hydrochloride, low-glucose (5.5 mM) Dulbecco's modified Eagle's medium (DMEM), high-glucose (25 mM) DMEM, and penicillin-streptomycin solution (×100) were purchased from FUJIFILM Wako Pure Chemical (Osaka, Japan). Fetal bovine serum (FBS) was obtained from Biowest (Nuaillé, France); 2,5-diphenyl-2H-tetrazolium bromide (MTT) was obtained from Nacalai Tesque (Kyoto, Japan).

The murine HCC cell line Hepa1-6 (RIKEN BioResource Research Center, Ibaraki, Japan) was cultured in low-glucose DMEM supplemented with 10% FBS and 1× penicillin/streptomycin at 37°C and 5% CO_2_. DMEM was prepared at various glucose concentrations by mixing low- and high-glucose DMEM in different proportions. To initiate the experiments, Hepa1-6 cells were seeded and incubated overnight in a low-glucose medium. The cells were then treated with a fresh medium, either without or with 2.5- or 5-mM GlcN, at various glucose concentrations (5.5–20.0 mM) for 6–72 h at 37°C and 5% CO_2_. The concentration of GlcN was based on previous in vitro studies [7]. For cultures lasting longer than 24 h, the medium was replaced every 24 h with a fresh medium containing either GlcN or PBS as the vehicle control.

### 2.2. MTT Assay

The MTT assay was performed to determine the cell growth rate. Hepa1-6 cells were seeded into 24-well plates at 1.25 × 10^4^ cells/cm^2^ and incubated for 24, 48, or 72 h. At each time point, bright field images of the cells were acquired using a BZ-X710 microscope (KEYENCE, Osaka, Japan) with a 20× objective lens. The media were then replaced with low-glucose DMEM containing MTT (0.5 mg/mL). After an additional 3 h, to dissolve any formazan crystals, the medium was removed, and 250 μL of dimethyl sulfoxide was added to each well. Absorbance at 570 nm was measured using a microplate reader (Multiskan FC; Thermo Fisher Scientific JP, Tokyo, Japan).

### 2.3. DNA Fragmentation

Hepa1-6 cells were seeded into 6-cm dishes at 1.25 × 10^4^ cells/cm^2^ and incubated for 24, 48, or 72 h with or without 5-mM GlcN. After incubation, cells were collected, pelleted through centrifugation at 3000 × g for 5 min, washed once with 1× PBS, and then lysed in cold lysis solution (0.15-M NaCl, 10-mM Tris HCl [pH 8.0], 10-mM EDTA2Na, and 0.1% sodium dodecylsulfate (SDS)). The lysates were then treated with proteinase K (0.1 mg/mL) at 55°C for 60 min. Subsequently, lysates were extracted twice using phenol-chloroform-isoamyl alcohol (FUJIFILM Wako Pure Chemical). DNA was precipitated by centrifugation with 2.5 volumes of 100% ethanol, rinsed with 70% ethanol, and resuspended in 10-mM Tris–EDTA buffer. DNA was then subjected to electrophoresis on 1.5% agarose gels to identify DNA fragmentation.

### 2.4. Real-Time PCR Analysis

Total RNA was extracted from cells seeded into 6-well plates with RNAiso Plus reagent (Takara Bio, Shiga, Japan). The RNA concentration was determined with a SimpliNano spectrophotometer (GE Healthcare Bioscience, Tokyo, Japan). Next, 1 μg of total RNA in a 10-μL reaction mixture was reverse transcribed into cDNA using ReverTra Ace qPCR RT Master Mix (TOYOBO, Osaka, Japan). For real-time PCR, each target gene was amplified using TB Green *Premix Ex Taq* II (Takara Bio) on a Thermal Cycler Dice Real-Time System (Takara Bio). The thermal regime for amplification was as follows: initial denaturation at 95°C for 30 s followed by 40 cycles of denaturation for 5 s at 95°C and annealing/extension for 30 s at 60°C. The glyceraldehyde-3-phosphate dehydrogenase (*GAPDH*) gene was used as the reference gene. The primers used for this reaction are listed in Supporting Table 1.

### 2.5. Western Blotting

Hepa1-6 cells treated in a 6-well culture plate were washed and lysed with 50 μL of 50-mM HEPES-buffered saline (pH 8.0) containing 1-mM EDTA, 1% Triton X-100, 0.45% sodium pyrophosphate, 2-mM Na_3_VO_4_, 100-mM NaF, and a cocktail of protease inhibitors (cOmplete; Sigma-Aldrich, Tokyo, Japan). Following lysis, the mixture was centrifuged at 13,000 × g and 4°C for 15 min. The protein concentration in the supernatant was measured using a BCA Protein Assay Kit (Takara Bio). An equal amount of protein (10 μg per lane) was separated onto a 15% acrylamide gel to measure the levels of LC3B proteins and a 10% acrylamide gel for the other proteins with SDS polyacrylamide gel electrophoresis (SDS-PAGE) (100 V for 30 min and 150 V for 1 h) and transferred to a Hybond P polyvinylidene fluoride (PVDF) membrane (GE Healthcare Bioscience). The membranes were blocked with a blocking reagent (Blocking One-P; Nacalai Tesque) for 60 min at room temperature (18°C–22°C) and then incubated at 4°C overnight with a primary antibody against each of the following: LC3B (#2775), p70 S6 kinase (p70S6K) (#9202), phosphorylated-p70S6K (#9205), Akt (#9272), phosphorylated-Akt (#9271), AMP-activated protein kinase (AMPK) (#2532), and phosphorylated AMPK (#2535), all purchased from Cell Signaling (Danvers, MA, USA), or *β*-actin (#sc47778) from Santa Cruz Biotechnology (Dallas, TX, USA). The membranes were then washed and incubated with horseradish peroxidase-conjugated secondary antibodies for 60 min at room temperature. Chemiluminescence of specific proteins was detected with the ECL Prime kit (GE Healthcare Bioscience). Image acquisition and quantification were performed using the iBright imaging system (Thermo Fisher Scientific JP).

### 2.6. Analysis of ATP Production and Glycolytic Capacity

Cellular ATP production and glycolytic capacity were evaluated using the glycolysis/OXPHOS Assay Kit (G270; Dojindo Laboratories, Kumamoto, Japan). Cells were seeded into a 96-well culture plate. During 24 h of incubation, 2.5-μM oligomycin (an inhibitor of the mitochondrial respiratory chain) was added to some wells for the last 3 h. To evaluate ATP production, an ATP working solution was added to each well, and the culture was incubated for 10 min at 25°C. Thereafter, the relative ATP content was measured as luminescence intensity using a multimode plate reader (EnSpire; PerkinElmer Japan, Yokohama, Japan). To evaluate glycolytic capacity, a 10× dilution of the culture supernatant (20 μL) was transferred to a 96-well assay plate and incubated with an 80-μL lactate working solution at 37°C for 30 min. Absorbance at 450 nm was determined to evaluate the relative lactate production—representative of glycolytic capacity—using the Multiskan FC microplate reader (Thermo Fisher Scientific JP).

### 2.7. Statistical Analysis

Data analysis was performed using the SPSS v24 software (IBM, Tokyo, Japan). For comparisons between two groups, unpaired Student's t-tests were applied. Two-way analysis of variance (ANOVA) was used to evaluate interactions in tandem with Fisher's post hoc test. The level of statistical significance was set at *p* < 0.05.

## 3. Results

### 3.1. GlcN Inhibits Cell Proliferation in Hepa1-6 Cells

**Figure 1:**
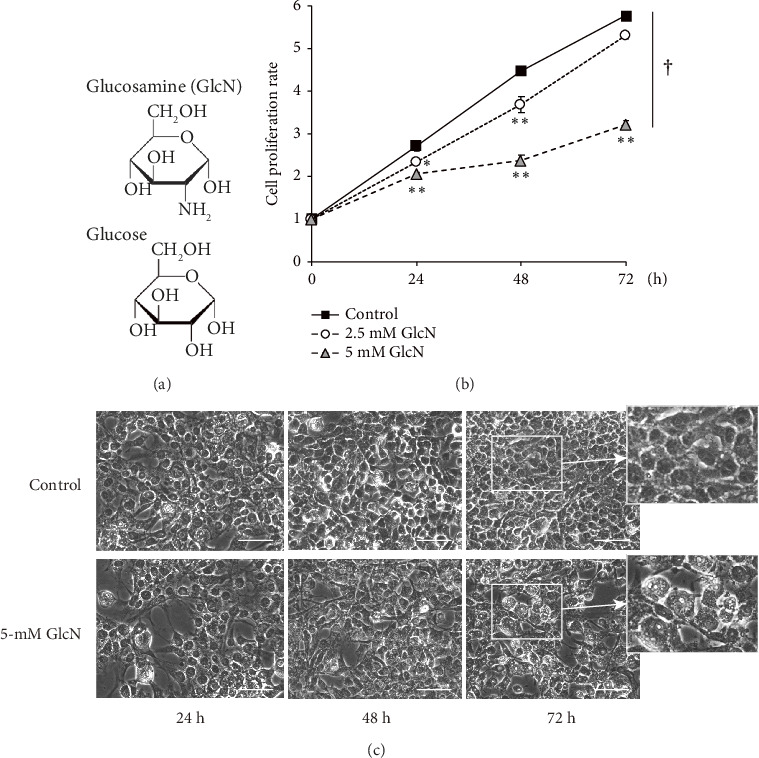
(a) Structures of glucose and glucosamine (GlcN). (b) Antiproliferative effect of GlcN on Hepa1-6 cells treated with 2.5- or 5.0 mM GlcN for 72 h versus those treated with a vehicle control. Cell proliferation rate was determined every 24 h via MTT assays; values are expressed as the fold changes from values recorded at the start of experiments (0 h), which were arbitrarily set to 1. Data are provided as the mean ± SE (*n* = 4). ⁣^∗^*p* < 0.05 and ⁣^∗∗^*p* < 0.01: significance versus vehicle-treated controls at the same time point; ^†^*p* < 0.05: significant interactions between groups. (c) Representative microscopic images of cultured cells at each time point without (control) or with 5 mM GlcN. Scale bar: 100 μm.

The chemical structures of GlcN and glucose are shown in Figure 1(a). To assess the biological role of GlcN in Hepa1-6 cells, we investigated its effect on cell proliferation. Our MTT assay revealed that cell growth was inhibited following cell incubation with GlcN (Figure 1(b)). The cell viability decreased to a viable cell rate of approximately 80% of that of the controls after 24 and 48 h of incubation with 2.5-mM GlcN. When the cells were treated with 5-mM GlcN, cell viability was significantly decreased at all time points, with ultimate viability being approximately half that of the untreated controls. After treatment with 5-mM GlcN for 48 and 72 h, the cells also exhibited morphological changes such as vacuolation (formation of vacuoles or vacuole-like structures within cells; Figure 1(c)), which marks cytopathological conditions leading to cell death [23, 24]. Thus, a 5-mM GlcN concentration was selected for use in further experiments.

### 3.2. GlcN Treatment Increased Proapoptotic Gene Expression

**Figure 2:**
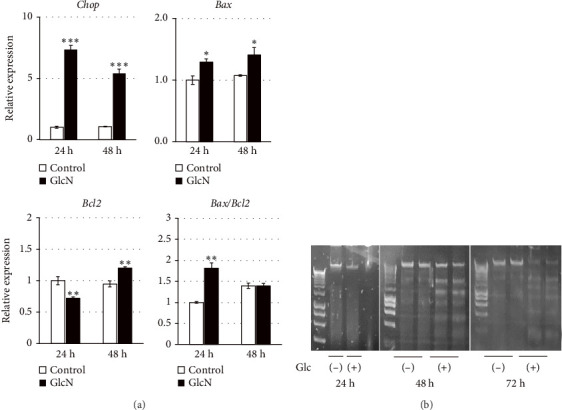
(a) Apoptosis-related gene expression in Hepa1-6 cells that were incubated without or with 5 mM GlcN for 24 and 48 h. The relative mRNA expressions of the *Chop*, *Bax*, and *Bcl2* genes were quantified; *Gapdh* served as a reference gene. Values are expressed as the fold change compared with the value from an untreated control after 24 h treatment, which was arbitrarily set to 1. Values represent the mean ± SE (*n* = 4). ⁣^∗^*p* < 0.05, ⁣^∗∗^*p* < 0.01, and ⁣^∗∗∗^*p* < 0.001 versus the control. (b) DNA fragmentation of Hepa1-6 cells after incubation for 24, 48, and 72 h in the absence (−) or presence (+) of GlcN.

Following GlcN treatment, we analyzed the expression levels of genes related to cell death (Figure 2). The expression of *Chop*, which encodes a well-known transcription factor that mediates endoplasmic reticulum (ER) stress-induced apoptosis, was markedly elevated in GlcN-treated cells after 24 h of incubation (Figure 2(a)). The expression of the proapoptotic gene *Bax* was also significantly increased, whereas that of the antiapoptotic gene *Bcl2* was significantly decreased after 24 h of GlcN treatment, thereby shifting the *Bax*/*Bcl2* ratio toward one promoting apoptotic changes (Figure 2(a)). *Chop* and *Bax* expression remained upregulated after GlcN treatment for 48 h (Figure 2(a)). However, the expression of *Bcl2* shifted from inhibited to enhanced, resulting in no significant difference in the *Bax*/*Bcl2* ratio between untreated and GlcN-treated cells. Consistently, DNA fragmentation, a specific marker of apoptosis, became evident after 48 h of incubation in GlcN-treated cells (Figure 2(b)).

### 3.3. GlcN Induces Energy Depletion and Autophagy

**Figure 3:**
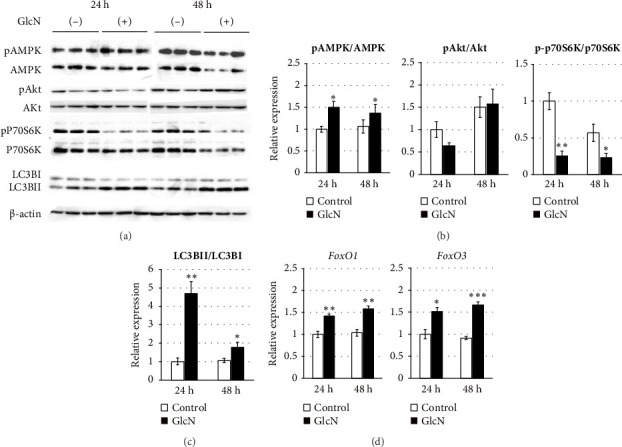
Gene and protein expression in Hepa1-6 cells incubated in the absence (−) or presence (+) of 5 mM GlcN for 24 and 48 h. (a) Representative Western blotting images are shown. Beta actin (*β*-actin) served as a loading control. (b) Protein phosphorylation levels of AMPK, Akt, and p70S6K as analyzed via Western blotting. (c) Protein expression of LC3BI and LC3BII as per Western blotting. (d) Relative mRNA expressions of *FoxO1* and *FoxO3* genes were quantified; *Gapdh* served as a reference gene. Values are expressed as the fold change compared with the value from an untreated control (−), which was arbitrarily set to 1. Values are presented as the mean ± SE (*n* = 3-4). ⁣^∗^*p* < 0.05, ⁣^∗∗^*p* < 0.01, and ⁣^∗∗∗^*p* < 0.001 versus the control.

We speculated that the GlcN-induced inhibition of cell proliferation could be partly attributed to insufficient intracellular energy. Therefore, we analyzed the activity of signaling proteins that reflect cellular energy status. The activity of AMPK, a key sensor of cellular energy depletion, was increased after 24 and 48 h of GlcN treatment (Figures 3(a) and 3(b)). In contrast, at the same time point, GlcN-treated cells exhibited a marked decline in the activity of p70S6K, which regulates cell growth via the induction of protein synthesis and is suppressed by energy deficiency (Figures 3(a) and 3(b)). Akt activation, which also regulates protein synthesis, was not observed (Figures 3(a) and 3(b)).

In eukaryotic cells, AMPK activity modulates autophagic degradation [25]. Consistent with the aforementioned finding of AMPK activation, cellular autophagy was also increased by GlcN treatment, as assessed by the observed LC3BII/LC3BI protein expression ratio after 24 and 48 h of GlcN treatment (Figures 3(a) and 3(c)). We also examined the expression of *FoxO1* and *FoxO3*, which encode forkhead box class O transcription factors involved in hepatic cell apoptosis and autophagy [26]. At 24 and 48 h, the expression of *FoxO1* and *FoxO3* was significantly upregulated by GlcN (Figure 3(d)).

### 3.4. Inhibition of Cell Proliferation by GlcN Treatment Is Restored via High Glucose Concentrations

**Figure 4:**
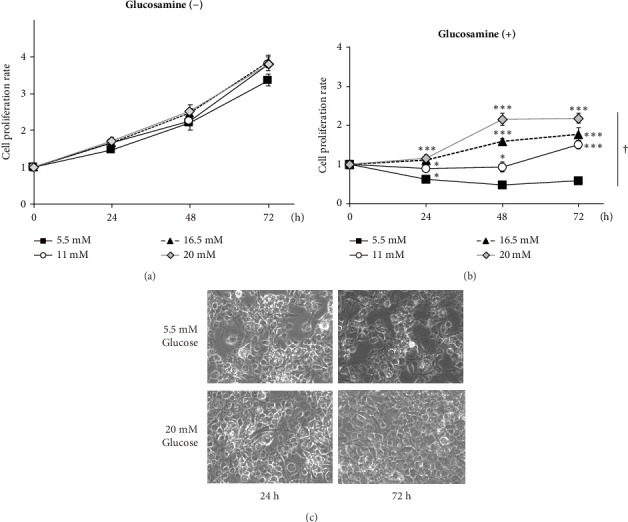
Effects of media glucose concentrations on the GlcN-induced inhibition of cell growth in Hepa1-6 cells. Cells were incubated with glucose at standard (5.5 mM) to high (11–20 mM) concentrations for 24, 48, and 72 h in the (a) absence or (b) presence of 5 mM GlcN. The cell proliferation rate was determined every 24 h via MTT assays. Values are expressed as the fold changes from values recorded at the start of experiments (0 h), which were arbitrarily set to 1. Data are given as the mean ± SE (*n* = 4). ⁣^∗^*p* < 0.05, ⁣^∗∗^*p* < 0.01, and ⁣^∗∗∗^*p* < 0.001 versus the control incubated with a standard glucose concentration (5.5 mM) at the same time point. ^†^*p* < 0.05: significant interactions between groups. (c) Microscopic images of GlcN-treated cells after incubation for 24 and 72 h with 5.5- or 20 mM glucose are shown.

GlcN has a high affinity for GLUT2 [21], a major isoform of the GLUT in the liver. Thus, we hypothesized that GlcN competes with glucose for uptake into hepatocytes, thereby inhibiting cell growth via energy depletion. To evaluate this hypothesis, we measured the proliferation rate of cells in media containing different glucose concentrations (5.5–20.0 mM). We observed that the antiproliferative effect of GlcN was diminished at higher glucose concentrations. In a medium without GlcN, the cell proliferation rate was not affected by glucose concentration (Figure 4(a)). In contrast, in GlcN-treated cells, the cell proliferation rate was restored in a dose-dependent manner in media containing glucose concentrations higher than the physiological standard concentration (5.5 mM; Figure 4(b)). In microscopic images, the abnormal morphological changes and growth retardation of GlcN-treated cells in normal glucose medium appeared to be alleviated in high glucose medium (Figure 4(c)).

### 3.5. GlcN-Induced Proapoptotic Changes and Autophagy Are Inhibited by High Glucose Concentrations

**Figure 5:**
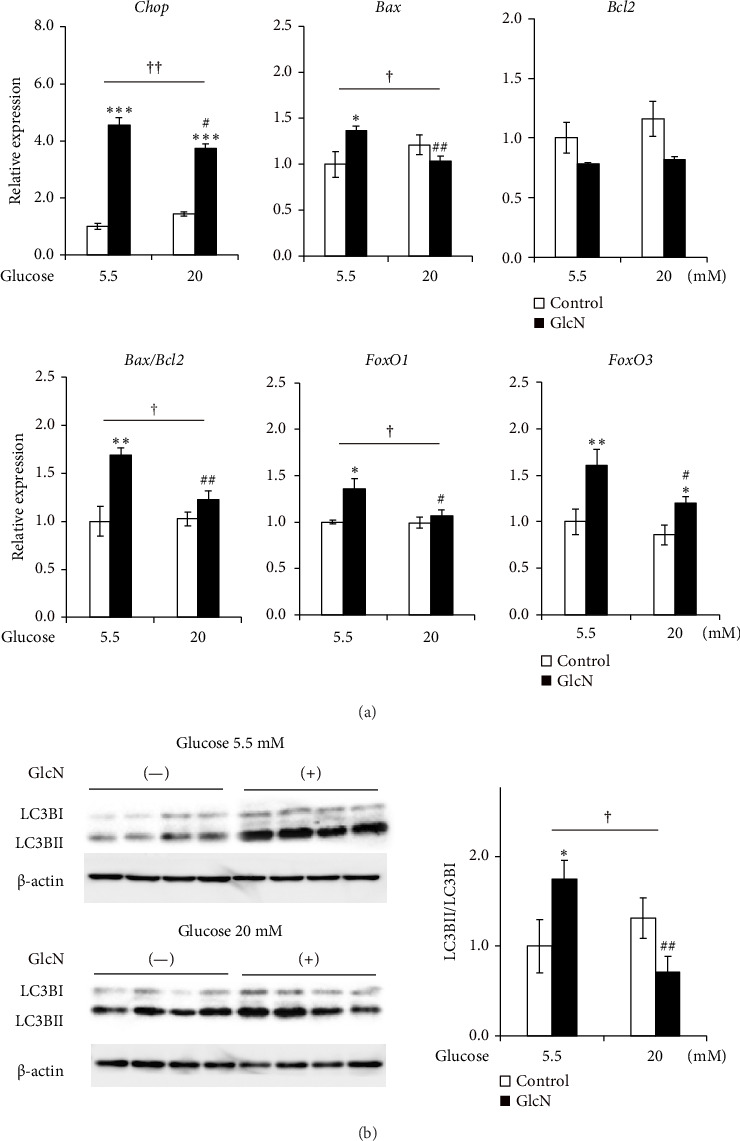
Effects of high glucose concentrations on GlcN-induced changes in gene expression and autophagic LC3II expression in Hepa1-6 cells. The cells were treated without (white bar) or with (black bar) 5 mM GlcN for 24 h in a medium containing standard (5.5 mM) or high (20 mM) glucose concentrations. (a) The relative mRNA expression of *Chop*, *Bax*, *Bcl2*, *FoxO1*, and *FoxO3* genes was quantified; *Gapdh* served as a reference gene. (b) Protein expression of LC3BI and LC3BII as per Western blotting. Representative blots are shown in the left-hand panel, and the LCB3II/LCB3I ratios are presented in the right-hand panel. Beta actin (*β*-actin) served as a loading control. Values are expressed as the fold change compared with values recorded for untreated cells in a medium with a standard glucose concentration, arbitrarily set to 1. Values are expressed as the mean ± SE (*n* = 4). ⁣^∗^*p* < 0.05 and ⁣^∗∗^*p* < 0.01 versus each control. ^#^*p* < 0.05 and ^##^*p* < 0.01 versus the group treated with GlcN in the medium with a standard glucose concentration. ^†^*p* < 0.05 and ^††^*p* < 0.01: significant interactions between groups.

The effects of high glucose concentrations on GlcN-induced changes in gene expression and autophagy were assessed. GlcN-induced increases in the expression of *Chop*, *Bax*, and *FoxO1* genes, along with the resulting increases in the *Bax*/*Bcl2* ratio, were significantly suppressed in cells maintained in a medium containing 20-mM glucose (Figure 5(a)). A similar suppressive trend was observed for the autophagy of cells in the same medium. As for protein expression, the GlcN-induced increases in the LC3BII/LC3BI ratio were mitigated by the treatment of cells with high glucose concentrations (Figure 5(b)).

### 3.6. GlcN Inhibits Glycolytic ATP Production, Which Is Restored by High Glucose Concentrations

**Figure 6:**
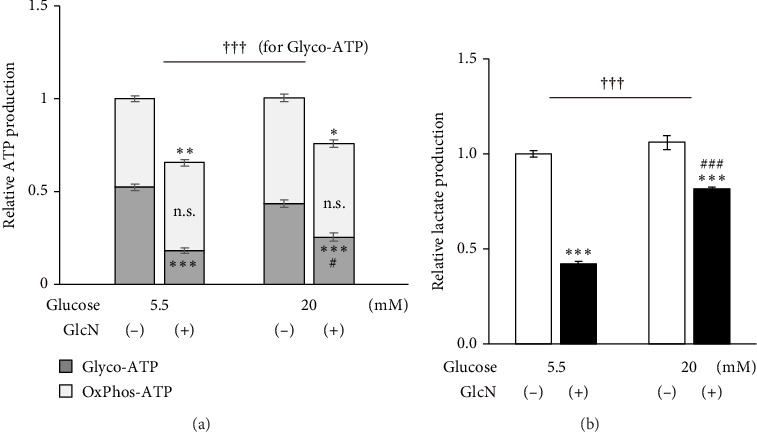
ATP and lactate production in Hepa1-6 cells incubated with different glucose concentrations. Cells were treated without (−) or with (+) 5 mM GlcN for 24 h in a medium containing either standard (5.5 mM) or high (20 mM) glucose concentrations. (a) ATP production rates stemming from glycolysis (Glyco-ATP) and oxidative phosphorylation (OxPhos-ATP) and (b) lactate production rates. Values are expressed as the fold change compared with the values of untreated cells in a standard glucose concentration (5.5 mM), which was arbitrarily set to 1. Values are given as the mean ± SE (*n* = 4). ⁣^∗^*p* < 0.05, ⁣^∗∗^*p* < 0.01, and ⁣^∗∗∗^*p* < 0.001 versus each control. ^#^*p* < 0.05 and ^###^*p* < 0.001 versus the group treated with GlcN in a normal medium. ^†††^*p* < 0.001: significant interactions between groups.

Finally, the effects of GlcN on cellular energy production were examined. Following the treatment of cells with GlcN for 24 h, there was a significant decrease in their total ATP production under both standard (5.5 mM) and high (20 mM) glucose conditions. However, this decline in ATP production was smaller in cells treated with high glucose concentrations, which exhibited a 35% decrease (from 1.00 to 0.65) under standard glucose conditions, as opposed to the 24% decrease (from 1.00 to 0.76) observed in cells under high glucose conditions (Figure 6(a)). Simultaneously, under respective standard versus high glucose conditions, glycolytic ATP production decreased from 0.52 to 0.18 and from 0.44 to 0.26, and the oxidative ATP production rate changed from 0.48 to 0.47 and from 0.57 to 0.50 (Figure 6(a)). These results indicate that the GlcN-induced decreases in ATP production were mainly caused by the inhibition of glycolytic ATP production, and this decrease was diminished by a higher glucose concentration. Similarly, intracellular lactate production—an indicator of aerobic glycolysis—was significantly decreased upon the treatment of cells with GlcN under both standard and high glucose conditions. The decline was significantly smaller in the cells cultured in a high-glucose medium, indicating that the GlcN-induced suppression of glycolysis was attenuated by higher concentrations of glucose (Figure 6(b)).

## 4. Discussion

Since the anticancer activity of GlcN was first reported by Quastel and Cantero in 1953 [5], many experimental and observational studies have been performed in this field. The results of large cohort studies have demonstrated a discernible relationship between GlcN usage and a reduced risk of lung, kidney, and colorectal cancer [8–10, 27]. Conversely, these investigations did not reveal any considerable association between GlcN usage and the amelioration of breast and urothelial carcinoma [11, 28–30]. Intriguingly, for prostate and skin cancers, the use of GlcN appeared to be correlated with an elevated risk [11]. These results indicate that the efficacy of GlcN may vary depending on the type of cancer. Consequently, various cell lines have been investigated to elucidate the anticancer mechanisms of GlcN [7]. However, scant information exists regarding the potential anticancer properties of GlcN in HCC. In this study, we demonstrated that GlcN exerts growth inhibitory effects in mouse HCC Hepa1-6 cells via multiple mechanisms.

Several anticancer mechanisms of GlcN have been reported, with its effect on ER stress and related cell death likely playing a role. GlcN affects protein N-glycosylation, which can induce ER stress [31, 32]. In turn, ER stress leads to the expression of several genes, including those encoding CHOP, which induces programmed cell death via the regulation of gene expression that favors apoptosis [33, 34]. Indeed, GlcN treatment has been demonstrated to cause ER stress-induced apoptosis and autophagic cell death in several carcinoma cell lines [35, 36]. In the present study, *Chop* expression was significantly upregulated by GlcN treatment, which was associated with the increased expression of proapoptotic *Bax* and the appearance of ladder DNA fragments in Hepa1-6 cells. These results suggest that ER stress-induced apoptosis may be partially involved in the inhibition of cell growth induced by GlcN treatment in this cell line. However, after 48 h of GlcN treatment, *Bcl2* mRNA expression increased, leading to a decreased *Bax/Bcl2* ratio, which is a representative marker of apoptosis. This finding indicates that the inhibition of cell proliferation, at least with prolonged GlcN treatment, may be attributed to apoptotic cell death along with other causes.

GlcN treatment led to the activation of AMPK, a sensor of cellular energy status, and cellular autophagy in the present study. Moreover, inactivation of p70S6K was observed in GlcN-treated cells. P70S6K is responsible for protein synthesis and cell growth, two major consumers of cellular energy, and is therefore inactivated in low-nutrient environments. In addition, the expression of *FoxO1* and *FoxO3* remained constantly enhanced by GlcN treatment. *FoxO1/3* are two of the four genes of FoxO (*FoxO1/3/4/6*), a subgroup of the forkhead box transcription factor family, that play a critical role in the hepatic adaptation to low-nutrient states [26]. FoxOs are activated by AMPK under fasting conditions and inactivated by insulin-Akt signaling under fed conditions [37, 38]. Activated FoxOs increase glucose production by enhancing the expression of genes related to hepatic gluconeogenesis [26] and further promote autophagy to adapt to low-nutrient states [39]. Cumulatively, these findings indicate that GlcN treatment leads to energy depletion in cells, which may inhibit Hepa1-6 growth through autophagy and the suppression of protein synthesis.

Moreover, increasing evidence has indicated that FOXO proteins possess anticancer properties. Evidence of FOXO acting as a negative regulator of tumor growth originated from a study on the nematode *Caenorhabditis elegans* [40] and was followed by many studies which supported the role of FOXO proteins as tumor suppressors in mammalian organs, including the liver [41–43]. FOXOs exert their antitumor effects through multiple mechanisms, including cell cycle blockade, enhancement of apoptosis and autophagy, and inhibition of tumor angiogenesis. Thus, as per our findings, GlcN-induced increases in the expression of *FoxO1/3* might be directly involved in the suppression of Hepa1-6 cell growth.

Under the assumption that energy depletion plays a role in the GlcN-induced inhibition of Hepa1-6 cell growth, we further examined potential mechanisms underlying this phenomenon. We discovered the suppression of the effects of GlcN in the presence of high glucose concentrations, suggesting competition between GlcN and glucose uptake into Hepa1-6 cells. We speculated that such competition occurs during the transportation of these compounds into hepatic cells via a GLUT, specifically GLUT2, which is abundantly expressed in the liver. Cancer cells rely primarily on glycolysis to fulfill their metabolic demands and consequently exhibit excess glucose requirements [44]. Indeed, in HCC tissue, glucose uptake is higher than in nontumoral liver tissue [45, 46]. Moreover, GLUT2 is highly expressed in liver tumors [19, 20], whereas the expression of GLUT1, which facilitates glucose uptake in a variety of carcinomas, is barely detectable in HCC and the HCC cell line HepG2 [20, 46–48]. These findings collectively suggest that glucose incorporation via GLUT2 is important for HCC growth. Of note, GLUT2 is a high-affinity GlcN transporter [21]; indeed, it exhibited a higher affinity for GlcN than for glucose at physiological concentrations (100 mg/dL = 5.5 mM). Thus, GlcN may inhibit glucose uptake into HCC cells as a competitor of GLUT2.

We also demonstrated that GlcN treatment decreased cellular ATP production in Hepa1-6 cells by suppressing glycolysis but not oxidative phosphorylation. The production of lactate, the end product of glycolysis, decreased in GlcN-treated cells. Indeed, the phenomenon of enhanced aerobic glycolysis, known as the Warburg effect, is a hallmark of malignant cancer and plays a pivotal role in cancer cell proliferation [44, 49–51]. This glycolytic phenotype provides a constant supply of metabolic intermediates essential for cell growth and is closely linked to tumor growth and invasion [51, 52]. Increased glycolysis has also been associated with adverse clinical outcomes in patients with HCC [53]. Thus, inhibition of glycolysis offers a promising strategy for novel HCC treatments, and anticancer strategies with an “anti-Warburg effect,” targeting key transporters involved in glycolysis, have already been considered. In this regard, several GLUT inhibitors have been evaluated for their benefits in HCC treatment; however, mainly GLUT1 has been targeted, as it is generally overexpressed in cancer [54–56]. Therefore, our finding that GLUT2 may affect the potential mechanism underlying GlcN action in Hepa1-6 cells could herald a new therapeutic approach that utilizes the unique metabolism of HCC.

Our study was limited in that we only employed in vitro experiments using a cell line. Cell lines are sometimes not representatives of the original cancer features [57]. Thus, further in vivo studies using a model of HCC are needed in the future.

## 5. Conclusions

The present study revealed that GlcN inhibited cell growth of the HCC cell line Hepa1-6. In this study, we propose a mechanistic model of the antitumor effects of GlcN on HCC as follows: GlcN exerts a toxic effect by directly or indirectly inducing ER stress and related apoptosis as well as autophagic cell death in hepatoma cells. In addition, GlcN increases the expression of *FoxO1* and *FoxO3*, which play a crucial role as tumor suppressors in various cancers. Finally, GlcN elicits anticancer properties through an anti-Warburg effect via the blockade of glycolytic flux, potentially by competing with glucose for intracellular uptake through GLUT2. Orally administered GlcN accumulates in the liver; therefore, the potential effects of GlcN identified in this study could be translated into an effective therapeutic strategy against liver cancer.

## Data Availability

The data that support the findings of this study are available from the corresponding author upon reasonable request.

## Supporting Information

Supporting Table 1: Primer sequences used for real-time PCR analysis.
