# Supplementary material for: Glucosamine Inhibits the Proliferation of Hepatocellular Carcinoma Cells by Eliciting Apoptosis, Autophagy, and the Anti-Warburg Effect
Source: Scientifica (Cairo). 2025 Jan 8;2025:5685884. doi: 10.1155/sci5/5685884 (PMC11735062; doi:10.1155/sci5/5685884)
Supplement: Supporting Information — Additional supporting information can be found online in the Supporting Information section. [file 5685884.f1.docx]

|  | GeneBank ID | Position | Sequences |
| --- | --- | --- | --- |
| *Chop* | NM_009716.3 | F; 812-830  R; 911-892 | F; GCAAGGAGGATGCCTTTTC  R; GTTTCCAGGTCATCCATTCG |
| *Bax* | NM_007527.4 | F; 289-308  R; 461-442 | F; TGCAGAGGATGATTGCTGAC  R; GATCAGCTCGGGCACTTTAG |
| *Bcl2* | NM_009741.5 | F; 1923-1942  R; 2050-2031 | F; CCTGTGGATGACTGAGTACC  R; GAGACAGCCAGGAGAAATCA |
| *FoxO1* | NM_019739.3 | F; 1390-1409  R; 1503-1486 | F; ACATTTCGTCCTCGAACCAG  R; GGATGGATACACCAGGGAATG |
| *FoxO3* | NM_001376967.1 | F; 1923-1941  R; 2070-2049 | F; TGCCTTGTCAAATTCTGTC  R; TGCACTAGCTGAATACAGTGAG |
| *Gapdh* | NM_001289726.2 | F; 830-848  R; 902-883 | F; GTCGTGGATCTGACGTGCC  R; ATGCCTGCTTCACCACCTTC |

Supplementary Table 1. Primer sequences used for real-time PCR analysis
